# Utilizing rare-earth doping and photoluminescence: La^3+^ co-doped SrMnO_3_ hetero structure nanocomposites for enhanced photocatalytic dye degradation

**DOI:** 10.1038/s41598-026-54073-3

**Published:** 2026-05-30

**Authors:** E. R. Sheha, Heba M. El Sharkawy, Amina L. Mohamed, Hanaa Selim

**Affiliations:** 1 Experimental Nuclear Physics Department, Nuclear Research Center, Egyptian Atomic Energy Authority, 13759 Nasr City, Egypt; 2 Cyclotron Facility, Egyptian Atomic Energy Authority, Cairo13759, Nasr City, Egypt; 3 Department of Analysis and Evaluation, Egyptian Petroleum Research Institute, Nasr City, 11727 Cairo Egypt; 4 National Research Centre, Textile Research and Technology Institute, Pretreatment and Finishing of Cellulose-based Fibre Department, 33 El-Behouth St. (former El-Tahrir str.), Dokki, P.O 12622, Giza, Egypt

**Keywords:** La,Sr co-doping, MnO heterostructure, visible-light photocatalysis, methylene blue degradation, charge separation, rare-earth doping, wastewater treatment, Chemistry, Environmental sciences, Materials science, Nanoscience and technology

## Abstract

In this study, La co-doped SrMnO₃ (LSM) perovskite nanomaterials with varying La contents (0.0–2.0 wt%) were synthesized via a solid-state method and evaluated for visible-light-driven photocatalytic degradation of methylene blue (MB). Structural characterization using X-ray diffraction (XRD) confirmed the formation of a single-phase perovskite structure, while transmission electron microscopy (TEM) revealed nanoscale morphology with reduced particle size upon optimal La incorporation. Surface functional groups were analyzed using Fourier-transform infrared spectroscopy (FTIR). Optical investigations by ultraviolet–visible diffuse reflectance spectroscopy (UV–Vis DRS) demonstrated a slight band gap narrowing from 2.38 to 2.24 eV, enhancing visible-light absorption, while photoluminescence (PL) analysis confirmed significant suppression of electron–hole recombination, particularly for the LSM3 sample (1.0 wt% La). The LSM3 photocatalyst exhibited the highest degradation efficiency (~ 99.03%) and rate constant (0.01648 min⁻¹), significantly outperforming pristine SrMnO₃ (~ 85.61%). Band edge analysis revealed that the valence band is sufficiently positive for hydroxyl radical (•OH) generation, whereas the conduction band is not favorable for direct superoxide formation. Accordingly, the photocatalytic process is governed by a defect-mediated charge transfer mechanism, in which La-induced oxygen vacancies and localized electronic states act as electron traps, enabling stepwise reduction of oxygen to generate reactive oxygen species. Radical scavenger experiments confirmed that •OH and •O₂⁻ are the dominant active species. The enhanced photocatalytic activity is attributed to the synergistic effects of defect-induced charge separation, reduced crystallite size (~ 9 nm), and internal electronic redistribution within the single-phase lattice. This work highlights an effective defect-engineering strategy for developing efficient visible-light photocatalysts for wastewater treatment applications.

## Introduction

Water contamination by synthetic dyes is a critical global environmental issue due to their toxicity, carcinogenicity, chemical stability, and resistance to conventional treatment processes^1,2^. Methylene blue (MB) was selected as the model organic pollutant due to its widespread use and environmental relevance. MB is a cationic dye extensively utilized in textile, paper, and dyeing industries, and is commonly detected in industrial wastewater. Its presence in aquatic environments poses significant ecological and health risks due to its toxicity, persistence, and resistance to biodegradation. In addition, MB strongly absorbs in the visible region and exhibits high chemical stability in aqueous solutions, making it a suitable probe molecule for evaluating photocatalytic activity under visible-light irradiation. Furthermore, in this study, MB is used as a benchmark pollutant due to its well-established degradation pathways and kinetics, enabling reliable comparison with reported photocatalytic systems. Recent studies have also highlighted the environmental impact of dye pollutants and emphasized the need for efficient photocatalytic treatment technologies, further supporting the selection of MB as a representative contaminant in this work^3–5^. To address the persistent challenge of organic dye pollution, advanced oxidation processes (AOPs) have emerged as effective remediation technologies. Among them, semiconductor-based photocatalysis is recognized as a green, cost-effective, and sustainable approach capable of achieving complete mineralization of organic pollutants under light irradiation^6,7^. Despite these advantages, the practical application of photocatalysts remains limited by intrinsic drawbacks such as wide band gaps, weak visible-light absorption, and rapid recombination of photogenerated electron–hole pairs^6,8^.Transition-metal oxides have gained significant interest as photocatalysts due to their abundance, low cost, structural flexibility, and tunable optical and electronic properties^9^. In particular, strontium manganese oxide (Sr@MnO) exhibits promising visible-light photocatalytic activity owing to its favorable band structure, high redox potential, and chemical stability. However, its performance is still restricted by limited charge-carrier mobility and fast electron–hole recombination^10–13^.

Previous studies on Sr–Mn–O-based perovskites support their potential in photocatalysis^10,12–14^. However, recent literature underscores the growing importance of multi-metal co-doping in manganese-based materials to further enhance environmental remediation. For instance, recent studies on Sr-doped BiVO_4_^14^ systems and heterojunction engineering in SrFe_12_O_19_/NiO^15^ demonstrate how alkaline-earth metals effectively tune the electronic band structure. Similarly, the construction of heterojunctions and the integration of rare-earth elements like Ce or La have been shown to effectively suppress charge carrier recombination and induce defect-mediated catalytic pathways^16^. These advancements align with the synergistic La, Sr co-doping strategy employed in this study to optimize the photocatalytic performance of the MnO system.Rare-earth doping has been widely employed to further enhance photocatalytic performance. Rare-earth ions can introduce localized energy levels, promote charge separation, and extend visible-light absorption^17–19^. Among them, lanthanum (La³⁺) is particularly attractive due to its large ionic radius and distinctive electronic configuration. Incorporation of La³⁺ into Mn-based lattices can induce lattice distortion, create oxygen vacancies, and facilitate charge transfer pathways, thereby suppressing recombination and enhancing photocatalytic efficiency^20–25^.In this regard, An et al.^17^, found that the La³⁺ is a rare earth element that can separate charge carriers and also allow more light to be absorbed into the system by creating localized states for charge carriers. Zheng et al.^19^ discussed the role that La³⁺ plays when it is doped into the predictable crystal lattice of a perovskite structure, as it has a larger ionic radius than other rare earth elements, this will distort a lattice and create oxygen defects. The electronic and photocatalytic properties of Mn-based oxides were affected by La doping as shown by Katoch et al.^26^ and Viswanathan et al.^22^ The most relevant result, that supports the design, is Achary et al.‘s^24^ findings that La-Doped strontium manganite had increased photocatalytic activity towards decolorizing organic dyes.

Although mono-doping strategies (e.g., Sr or La individually) improve certain material properties, they generally address only one limitation at a time, leaving other intrinsic challenges insufficiently resolved. To overcome this issue, we propose a dual-modification strategy that integrates Sr@MnO heterostructure formation with optimal La doping (1.0 wt%). This co-doping approach simultaneously enhances charge separation through Sr-induced heterostructure effects and tunes the electronic structure via La³⁺-induced defect engineering. The synergistic interaction between Sr and La leads to improved visible-light absorption, reduced electron–hole recombination, and superior photocatalytic activity compared with previously reported mono-doped MnO systems.

In this work, a series of La co-doped SrMnO_3_ heterostructure nanocomposites were synthesized via a solid-state method and systematically characterized using X-ray diffraction (XRD), high-resolution transmission electron microscopy (HR-TEM), and UV–Vis diffuse reflectance spectroscopy (DRS). Their photocatalytic performance was evaluated through methylene blue degradation under visible-light irradiation. Particular emphasis was placed on correlating structural modifications, defect formation, and band-gap tuning with photocatalytic efficiency. This study provides new insight into the synergistic effects of rare-earth and alkaline-earth co-doping in MnO-based systems and offers a rational strategy for designing high-performance photocatalysts for wastewater treatment applications.

From a practical perspective, the scalability of La co-doped SrMnO_3_ photocatalysts is promising due to the simplicity and cost-effectiveness of the solid-state synthesis method, which can be readily extended to large-scale production. In addition, the use of abundant precursor materials and the excellent stability and reusability observed for the LSM3 sample support its feasibility for real wastewater treatment applications. For practical deployment, such photocatalysts can be utilized in slurry-type reactors or immobilized systems to facilitate catalyst recovery and reuse. However, further investigations on pilot-scale systems, long-term operational stability, and performance in complex wastewater matrices are still required. These considerations are consistent with recent studies highlighting the importance of catalyst stability, scalability, and reactor integration for real environmental applications.

## Experimental section

### Chemicals and materials

Analytical grade lanthanum nitrate hexahydrate [La(NO₃)₃·6 H₂O, ≥ 99% purity], strontium nitrate [Sr(NO₃)₂, ≥ 99% purity], and manganese(II) oxide [MnO, ≥ 99% purity] were used as starting precursors. All chemicals were purchased from Sigma-Aldrich (USA) and used without further purification. Ethanol (≥ 99.5%) and deionized water were employed as dispersing and washing agents during sample preparation. An agate mortar and pestle, along with a planetary ball mill, were used for grinding and homogenization of the powders.

La, Sr co-doped MnO heterostructure nanocomposites were synthesized via a conventional solid-state method. Lanthanum nitrate [La(NO₃)₃·6 H₂O], strontium nitrate [Sr(NO₃)₂], and manganese oxide (MnO) were used as starting precursors. The reagents were weighed according to the desired La: SM weight ratios of 0.0, 0.25, 0.5, 1.0, and 2.0 wt%, corresponding to samples denoted as SM, LSM1, LSM2, LSM3, and LSM4, respectively. The calculated amounts were thoroughly mixed using an agate mortar and pestle for 1 h to ensure compositional homogeneity, followed by ball milling for 6 h in ethanol as a dispersing medium. The resulting slurry was dried at 80 °C overnight to remove residual solvent, and the obtained powder was reground to achieve fine particle size. Subsequently, the dried powders were calcined in a muffle furnace at 800 °C for 4 h with a heating rate of 5 °C min⁻¹ to facilitate phase formation and crystallization of the LSM heterostructure nanocomposites. After calcination, the samples were allowed to cool naturally to room temperature, ground into fine powders, and stored in airtight containers for further characterization, as illustrated in Schematic 1. The selected dopant concentrations (0.25–2.0 wt% La) were based on recent optimization studies on rare-earth-doped perovskite systems. Previous reports indicate that La incorporation within the range of 0.5–2.0 wt% significantly enhances catalytic activity in Mn-based oxides, while an optimal loading of approximately 1.0 wt% yields superior photocurrent response and pollutant degradation efficiency. This range represents a balance between the beneficial effects of dopant-induced defect states and the detrimental formation of recombination centres at higher doping levels. The solid-state synthesis route was chosen due to its scalability, simplicity, and ability to produce well-crystallized materials with high reproducibility, without the need for complex solvent systems. Furthermore, the annealing temperature of 800 °C was selected based on prior studies of perovskite manganites, where temperatures in the range of 700–900 °C are sufficient to promote phase formation and crystallinity. This temperature provides adequate thermal energy for solid-state diffusion and lattice stabilization while minimizing excessive grain growth and the formation of secondary phases.

**Schematic 1:**
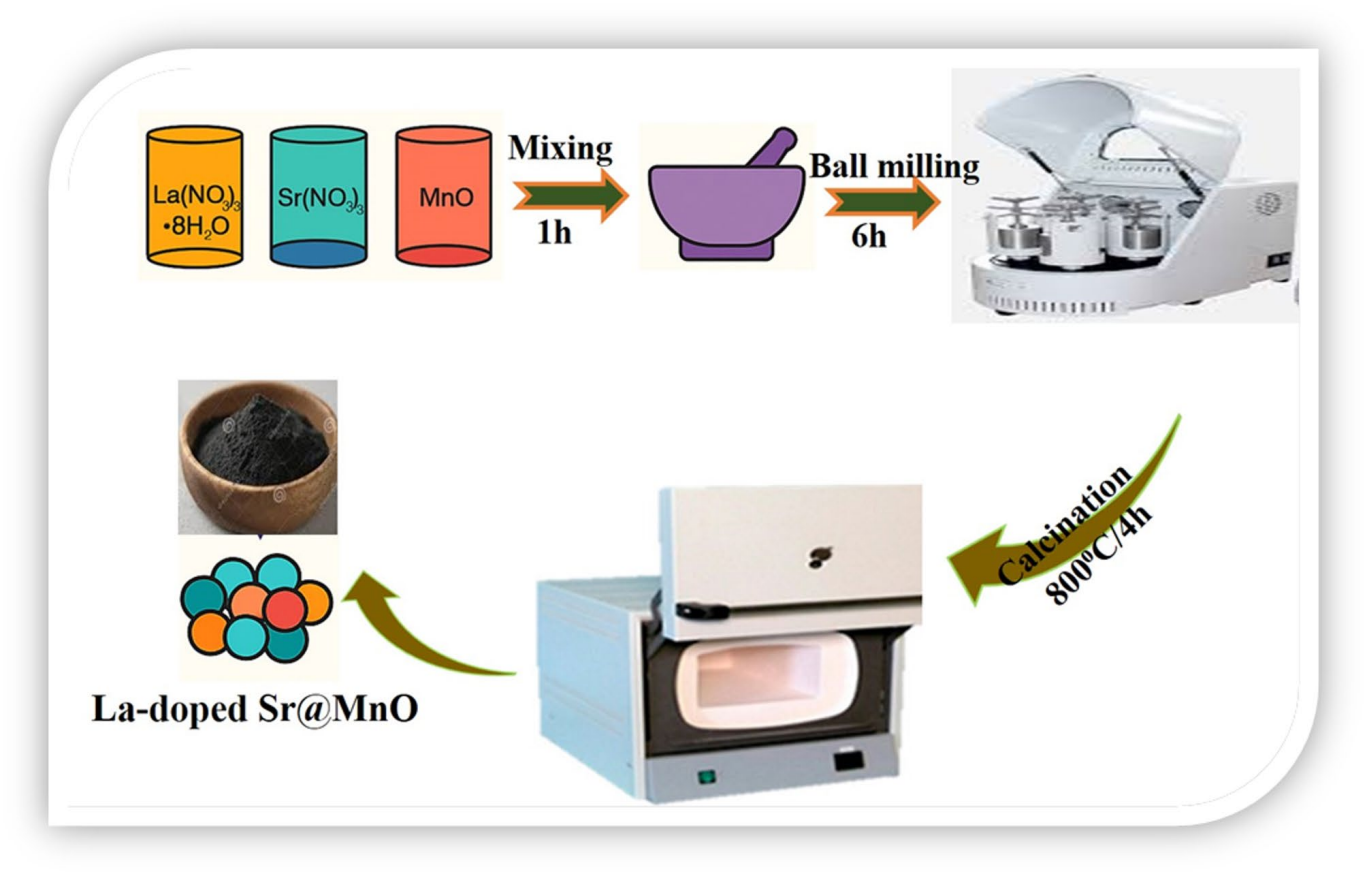
Solid –State synthesis of La, Sr co-doped MnO heterostructure (LSM) nanocomposite.

### Characterization techniques

The crystallographic structure of the synthesized heterostructure nanocomposites was analyzed using X-ray diffraction (XRD, Philips X’Pert) with Cu Kα radiation (λ = 1.54056 Å) operated at 40 kV and 40 mA, with a 2θ scan range of 10–80° at a rate of 4°/min. Fourier-transform infrared spectroscopy (FTIR, Nicolet iS10) was used to identify surface functional groups. Transmission electron microscopy (TEM, JEOL JEM 3200 FS, 300 kV) was conducted to investigate morphology. The optical properties were studied using UV–Vis spectroscopy (Jasco V-507) with diffuse reflectance (Shimadzu IRS-2200), while photoluminescence (PL) spectra were obtained using a Perkin Elmer LS-55 fluorometer at room temperature. 

### Photocatalytic activity study

The photocatalytic performance of the synthesized La co-doped SrMnO₃ heterostructure nanocomposites was investigated using methylene blue (MB) as a model organic pollutant under visible light irradiation. Experiments were conducted at room temperature with a 500 W halogen lamp positioned 10 cm from the reaction cell. For each test, 50 mL MB solutions of varying initial concentrations (20 ppm) were prepared, and the catalyst was added at a fixed dosage of 1.5 g/L. The suspensions were magnetically stirred in the dark for 30 min to establish adsorption–desorption equilibrium, followed by continuous stirring under visible light irradiation. At predetermined time intervals (30–210 min), 3 mL aliquots were collected, centrifuged to remove the catalyst, and analyzed using a UV–Vis spectrophotometer (UV-1800, Shimadzu, Japan) at λ_max = 622 nm. Control experiments without catalyst and under dark conditions revealed negligible MB degradation, confirming that both light irradiation and the catalyst are essential for efficient photocatalysis.

## Results and discussion

### Structural analysis

#### HR-TEM analysis

**Fig. 1:**
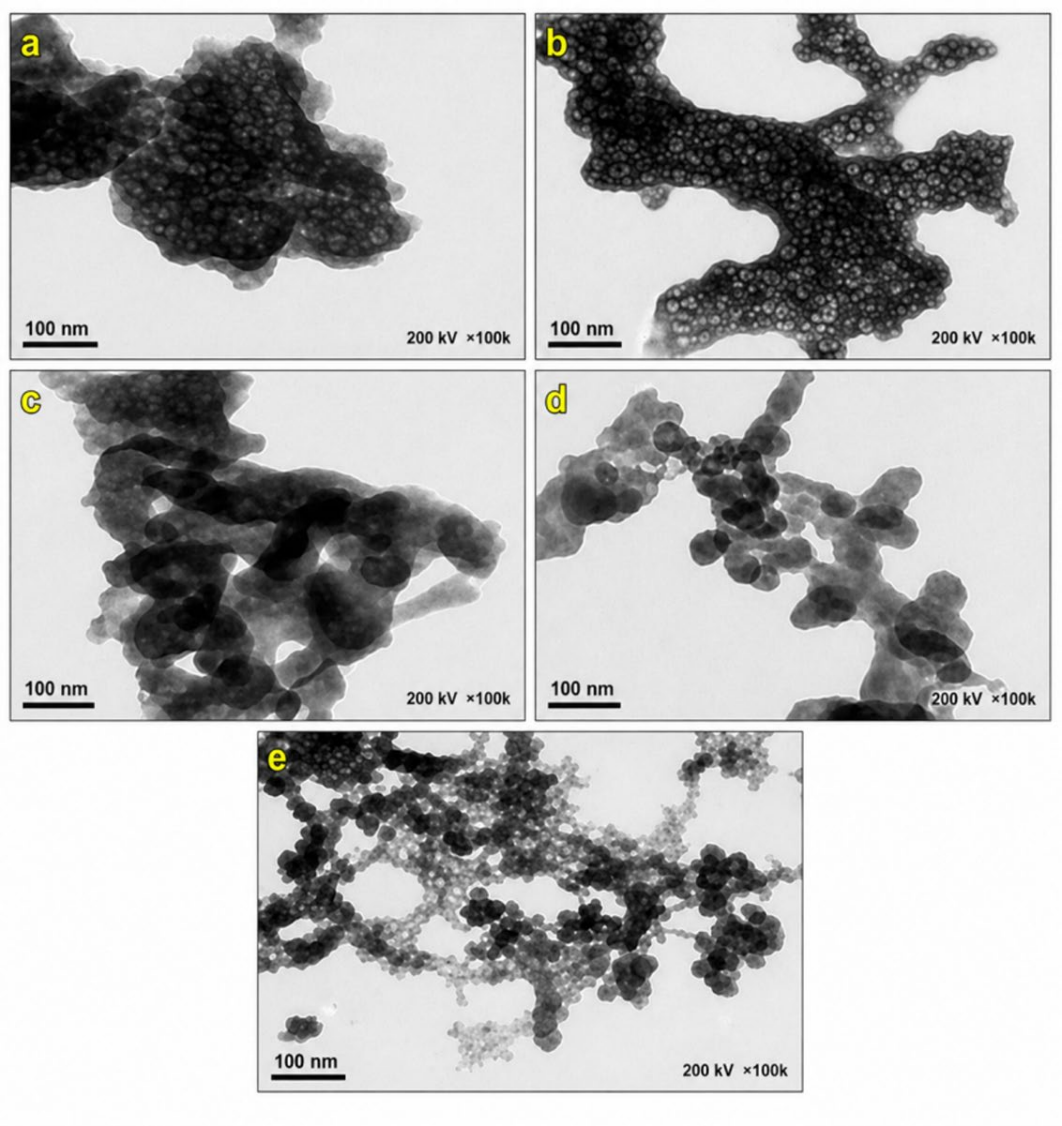
**(a-e)**: TEM Micrographs of SM and LSM (1–4) nanocomopsites, respectively.

Transmission Electron Microscopy (TEM) analysis (Fig. 1) was conducted to characterize the dopant-induced morphological evolution in Sr@MnO. The pristine Sr@MnO sample was composed of irregularly agglomerated, dense nanosheets, evidenced by dark Black stripes corresponding to overlapping layers. Introducing 0.25 wt% La reduced this agglomeration, yielding nanoparticles with more defined boundaries, suggesting that La^3+^ inhibited grain growth. At the optimal concentration of 0.5 wt% La, the nanoparticles became smaller, more compact, and uniformly dispersed, indicating that La^3+^ ions on the particle surface effectively stabilized the structures and minimized coalescence during synthesis. Further increasing the doping to 1 wt% La resulted in the most refined morphology, characterized by nearly spherical nanoparticles with excellent size uniformity and minimal agglomeration. This structural refinement is attributed to lattice distortion and the generation of conductive defects from La^3+^ substitution, which is expected to increase the density of active sites and surface area, thereby enhancing catalytic and electrochemical performance. However, at 2 wt% La doping, the material began to re-agglomerate and form irregular clusters, a result of elevated lattice strain and increased interparticle interactions expected at higher dopant concentrations^25,31,32^.

#### X-ray diffraction analysis (XRD)

**Fig. 2:**
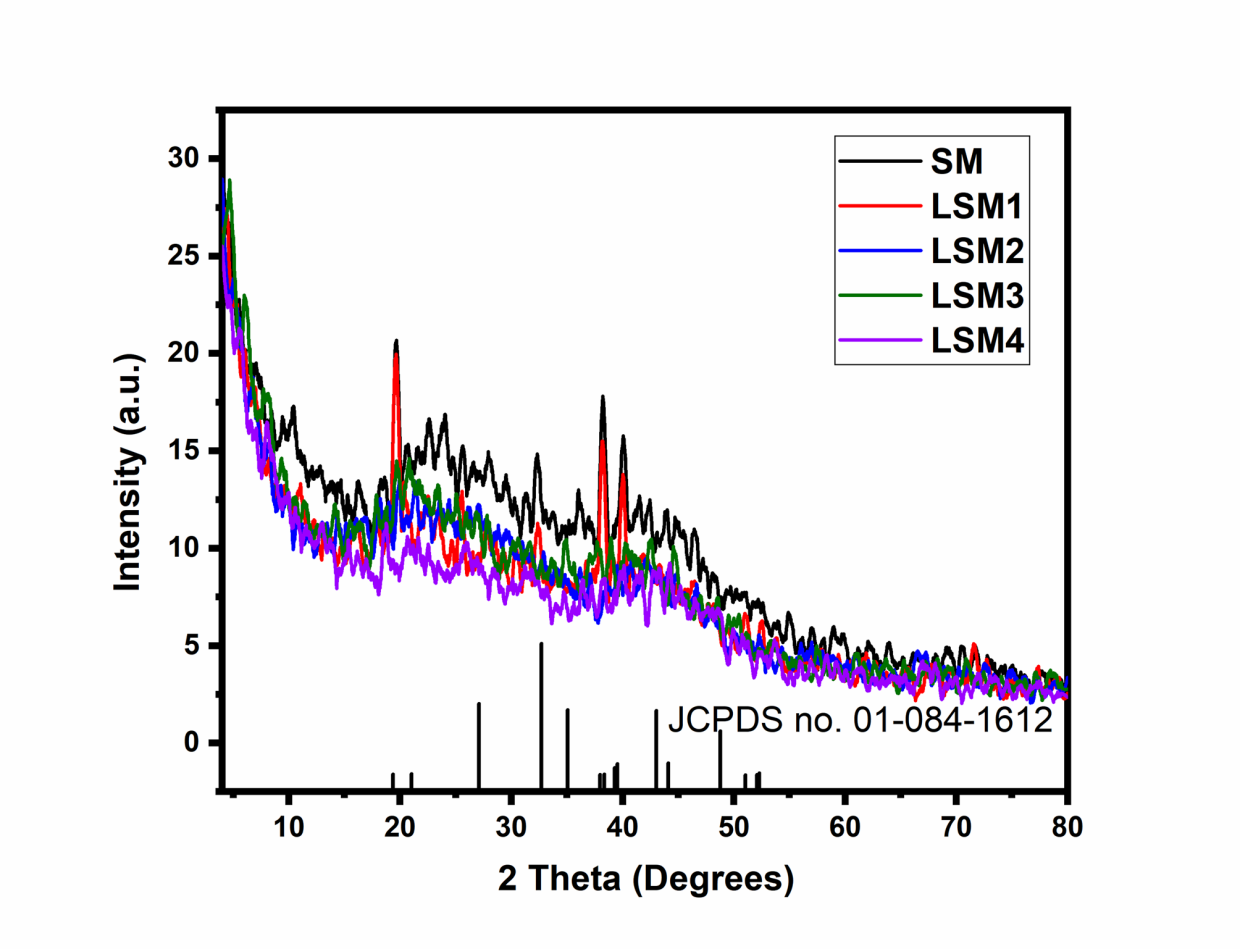
XRD pattern of SM and LSM nanocomopsites.

The crystalline structure and phase purity of the pristine and La-doped samples were investigated using XRD, as illustrated in Fig. 2. All diffraction peaks for the pristine sample (SM) and the La-doped series (LSM1–LSM4) are precisely indexed to the hexagonal perovskite phase of Strontium Manganate (SrMnO_3_), consistent with JCPDS card no. 84-1612. The XRD patterns exclusively match the hexagonal phase of SrMnO_3_ with no evidence of secondary oxide phases. Consequently, the material is classified as a single-phase doped perovskite. The enhanced performance is not due to a multi-phase heterostructure, but rather to lattice-strain-induced defects and a doping-gradient-driven charge separation mechanism. The characteristic reflections observed at 2Ө≈ 19.6^o^, 25.56^o^, 38.23^o^, 40.02^o^, and 52.46^o^ correspond to the (002), (101), (110), (103), and (202). crystal planes, respectively. Notably, no characteristic peaks corresponding to MnO or SrO were detected, confirming the formation of a high-purity, single-phase perovskite structure.The introduction of La^3+^ ions as a co-dopant significantly influences the structural parameters without altering the fundamental hexagonal symmetry. A detailed inspection of the most intense peaks reveals a systematic shift toward lower 2theta values with increasing La content. For instance, the (110) reflection shifts from38.23^o^ in the pristine SM to38.15^o^ in LSM1. This shift indicates an expansion of the unit cell volume, which is attributed to the substitution of Sr^2+^ ions (ionic radius ≈ 1.18 Å) byLa^3+^ions (ionic radius ≈ 1.03Å). Although La^3+^ is slightly smaller than Sr^2+^, the substitution often induces lattice distortion and changes in the Mn oxidation state Mn^4+^ to Mn^3+^ to maintain charge neutrality, leading to the observed lattice expansion.The average crystallite sizes were calculated using the Scherrer equation based on the FWHM of the primary diffraction peaks (Table 1). The pristine SrMnO_3_ (SM) exhibited a crystallite size of 15.12 nm. Upon doping, a notable refinement of the particle size was observed, with LSM3 (1.0 wt% La) reaching a minimum of 9.01 nm. This reduction in crystallite size is beneficial for photocatalysis, as it increases the specific surface area and provides more active sites for the degradation of Methylene Blue (MB). However, at higher doping concentrations (LSM4, 2.0 wt%), a decrease in peak intensity and an increase in FWHM were observed, suggesting a degree of lattice disorder or incipient segregation of La species, which may account for the slightly lower photocatalytic performance compared to LSM3.In summary, the XRD analysis confirms that the materials are La co-doped SrMnO_3_ perovskites. Moderate La incorporation (1.0 wt%) optimizes the structural properties by reducing crystallite size and inducing beneficial lattice strain, which collectively enhance the separation of photogenerated charge carriers^28,29,33,34^.

**Table 1:** **Crystallographic parameters of** La co-doped SrMnO_3_ **samples.**

Sample	Main Peak (2theta)	FWHM (deg)	Crystallite Size (nm)
SM	38.23^o^	0.5560	15.12 nm
LSM1	38.15^o^	0.5700	14.75 nm
LSM2	21.33^o^	0.4462	18.12 nm
LSM3	20.81^o^	0.8969	9.01 nm
LSM4	20.65^o^	0.5334	15.14 nm

The crystallite size plays a crucial role in governing charge carrier dynamics and, consequently, photocatalytic performance. As determined from XRD analysis, the LSM3 sample exhibits the smallest crystallite size (~ 9.01 nm), which is strongly correlated with its superior photocatalytic activity. The reduction in crystallite size increases the specific surface area, providing more active sites for dye adsorption and surface reactions. In addition, smaller crystallites shorten the diffusion path length of photogenerated electron–hole pairs, thereby minimizing bulk recombination and facilitating faster charge transfer to the catalyst surface. This interpretation is further supported by photoluminescence (PL) analysis, where LSM3 shows the lowest emission intensity, indicating suppressed recombination and enhanced charge separation efficiency. In contrast, at higher La content (LSM4), the slight increase in crystallite size and lattice disorder may introduce defect-induced recombination centres, leading to a marginal decline in photocatalytic performance. Therefore, the optimized crystallite size in LSM3 provides a balance between high surface reactivity and efficient charge carrier separation, which is essential for achieving enhanced photocatalytic efficiency.

#### FT-IR spectroscopy

**Fig. 3:**
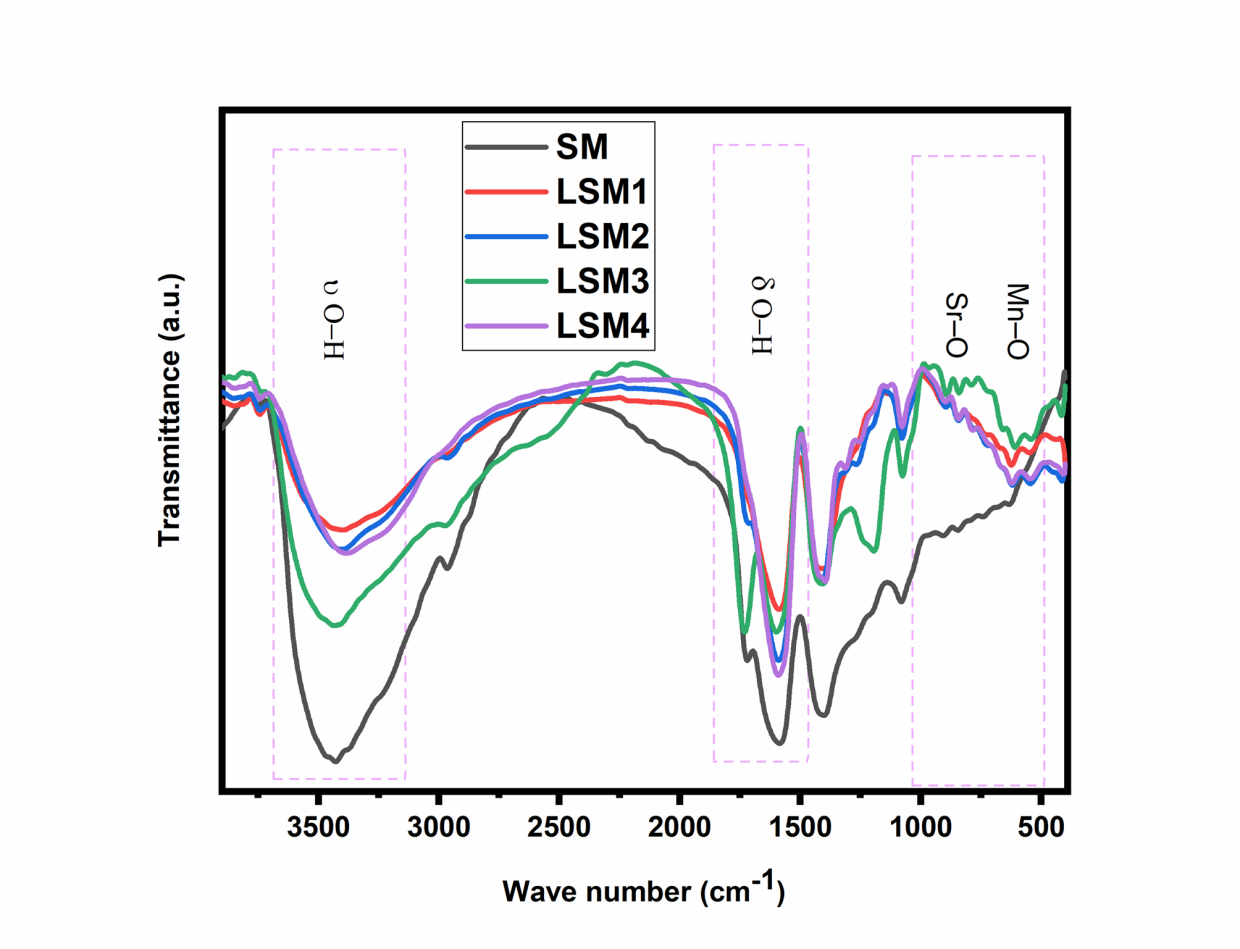
FT-IR spectra of SM and LSM nanocomopsites.

Figure 3 presents the FT-IR spectra of pristine Sr@MnO (SM) and La co-doped SrMnO₃ heterostructured nanocomposites (LSM1–LSM4) with La loadings of 0.25, 0.5, 1.0, and 2.0 wt%. All spectra exhibit characteristic absorption bands corresponding to metal–oxygen vibrations, confirming the formation of the mixed oxide framework. The broad band observed at 3400–3450 cm⁻¹ is attributed to the stretching vibration of surface –OH groups, originating from adsorbed moisture and hydroxyl species, which play a crucial role in photocatalytic reactions. A weaker band at ~ 1630–1640 cm⁻¹ corresponds to the bending vibration of H–O–H, further indicating the presence of physically adsorbed water^35^. The prominent absorption bands in the 500–650 cm⁻¹ region are assigned to Mn–O and Sr–O stretching vibrations, representing the fingerprint region of the perovskite-like Sr@MnO structure. Upon La incorporation, no new peaks appear, confirming that La³⁺ ions are successfully integrated into the host lattice without altering the fundamental structure. However, slight shifts and variations in band intensity are observed, particularly for LSM3 (1.0 wt% La), indicating lattice distortion and strengthened metal–oxygen interactions due to substitutional doping^36–39^. The gradual change in peak intensity with increasing La content suggests enhanced surface hydroxylation and modified bond strength, both of which are favorable for photocatalytic activity. Notably, LSM3 exhibits the most balanced spectral features, indicating optimal La incorporation that enhances surface reactivity while preserving structural integrity. Overall, the FT-IR results confirm that La incorporation modifies the local bonding environment, increases surface hydroxyl groups, and induces lattice distortion, collectively contributing to improved photocatalytic performance^40,41^.

### Optical analysis

The optical properties of the synthesized samples were examined using UV–Vis diffuse reflectance spectroscopy (DRS) and photoluminescence (PL) at room temperature.

#### DRS spectra and band gap

Photocatalytic activity is governed by several key factors, including light absorption, electron–hole (e⁻/h⁺) pair generation, charge separation, and charge utilization. Among these, the energy band gap (Eg) plays a critical role, as it determines the efficiency of photoinduced charge carrier excitation and transfer. The band gap energies of Sr@MnO (SM) and La co-doped SrMnO₃ heterostructure nanocomposites (LSM1–LSM4) were estimated from diffuse reflectance spectra (DRS) using the Tauc Eqs.^42–44^:

1 $$~\left( {\alpha {\mathrm{hv}}} \right)^{{\mathrm{n}}}={\text{ A}}\left( {{\mathrm{hv}} - {\mathrm{E}}_{{\mathrm{g}}} } \right)~~$$

Where α is the absorption coefficient, hν is the photon energy, A is a constant, and n represents the nature of the electronic transition. In this study, a direct allowed transition (*n* = 2) was adopted, consistent with reported perovskite-type oxides.

**Fig. 4:**
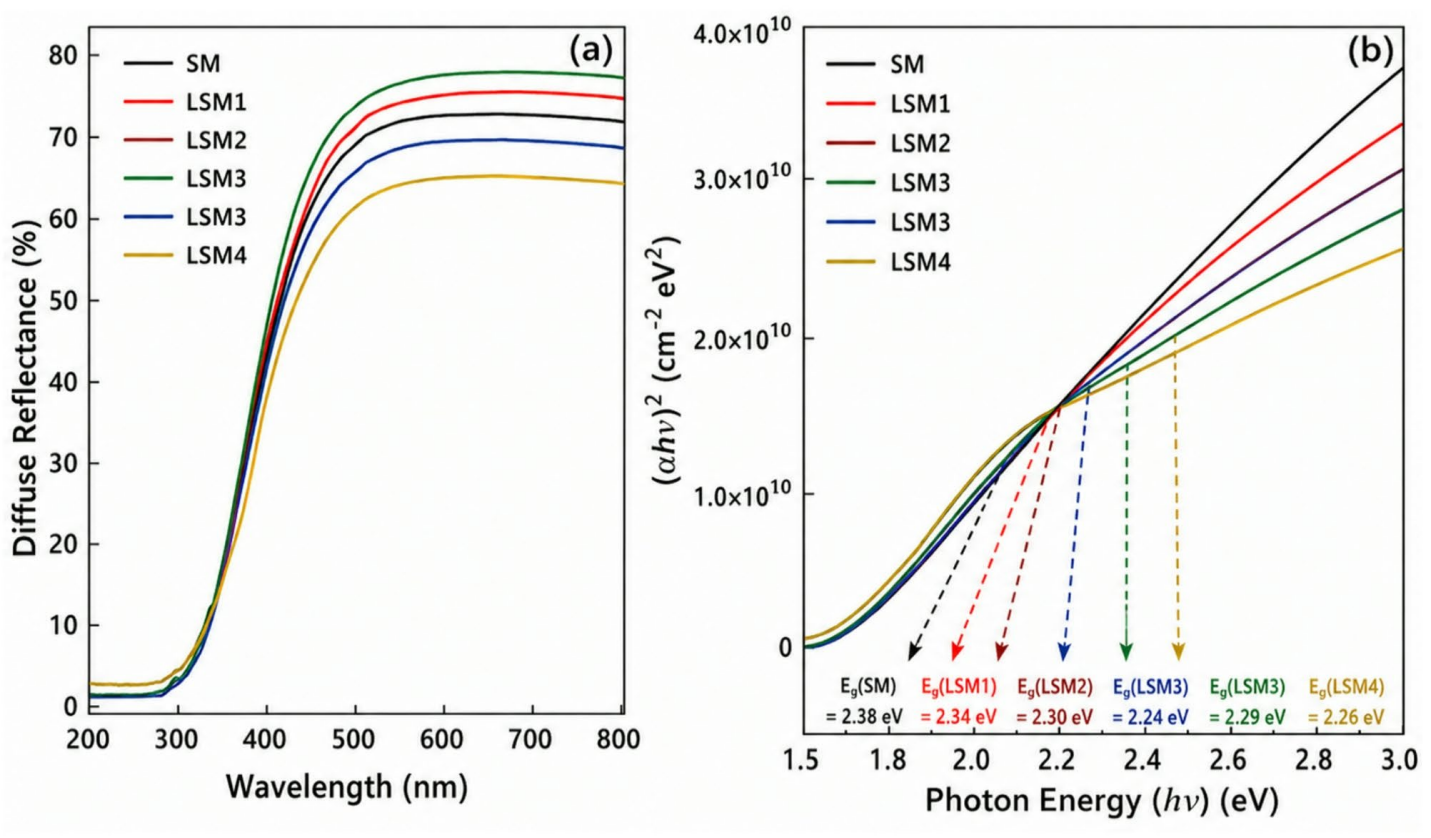
(**a**) UV–Vis diffuse reflectance spectra and (**b**) Tauc plot of LSM nanocomopsites.

Figure 4(a) shows the DRS spectra in the 200–800 nm range. Pristine SM exhibits strong absorption in the UV region (< 400 nm), mainly associated with defect-related transitions and Mn³⁺/Mn⁴⁺ intervalence charge transfer within the Sr–Mn–O lattice, rather than intrinsic band-gap narrowing. Upon moderate La incorporation (LSM1–LSM3), a distinct red shift in the absorption edge is observed, indicating improved visible-light absorption. This behavior is attributed to La³⁺-induced lattice distortion and the formation of shallow defect states, which modify the electronic structure and promote visible-light-driven excitation. Notably, LSM3 (1.0 wt% La) demonstrates the most favorable optical response, correlating well with its highest photocatalytic performance.In contrast, excessive La doping (LSM4, 2.0 wt%) results in a slight blue shift of the absorption edge and a marginal increase in band gap energy. This can be ascribed to increased lattice distortion and structural disorder, as supported by XRD analysis. Overdoping may suppress beneficial defect states while introducing recombination centers, thereby reducing visible-light absorption. Furthermore, a Burstein–Moss-type effect arising from increased carrier concentration may contribute to the apparent band-gap widening. In contrast, excessive La doping (LSM4, 2.0 wt%) leads to a slight blue shift and increased band gap due to lattice distortion and structural disorder, as confirmed by XRD analysis. Overdoping suppresses beneficial defect states and introduces recombination centers, reducing visible-light absorption. Furthermore, a Burstein–Moss-type effect arising from increased carrier concentration may contribute to the apparent band-gap widening.

The band gap values were derived from Tauc plots based on the Kubelka–Munk function, using only the linear region of the absorption edge for extrapolation. Both direct (*n* = 2) and indirect (*n* = 1/2) models were evaluated, with the direct transition providing the best linear fit (highest R²), in agreement with previous studies on Sr–Mn–O systems.

Although La doping leads to slight band-gap narrowing (from 2.38 to 2.24 eV), the variation remains modest and cannot solely explain the significant enhancement in photocatalytic activity as revealed in Table 2. This suggests that band-gap tuning plays a secondary role. The improved performance is primarily attributed to enhanced charge separation efficiency, as evidenced by photoluminescence quenching, along with La³⁺-induced defect states and oxygen vacancies that facilitate charge transfer and suppress electron–hole recombination. Additionally, morphological improvements and increased surface-active sites contribute to the superior activity of LSM3. Although band gap reduction improves visible light absorption, photocatalytic efficiency is also strongly governed by the recombination rate of electron–hole pairs. Efficient suppression of recombination is essential to fully utilize the generated charge carriers.

Overall, moderate La doping (LSM3) effectively optimizes the band structure and visible-light absorption, whereas excessive doping leads to slight band-gap widening and reduced photocatalytic efficiency^24,27,45–47^.

**Table 2:** Calculated band edge positions (vs. NHE) of SM and LSM nanocomopsites.

Sample	La wt% (added)	E_g_ (eV)	E_VB_ (V)	E_CB_ (V)
SM	0.0	2.38	+ 2.39	+ 0.01
LSM1	0.25	2.34	+ 2.37	+ 0.03
LSM2	0.50	2.30	+ 2.35	+ 0.05
LSM3	1.0	2.24	+ 2.32	+ 0.08
LSM4	2.0	2.29	+ 2.35	+ 0.06

#### Photoluminescence (PL) spectroscopy

The photoluminescence (PL) spectra provide valuable insight into the recombination behavior of photogenerated charge carriers (e⁻/h⁺). Generally, a higher PL intensity indicates a higher rate of electron–hole recombination, which is detrimental to photocatalytic performance, whereas lower PL intensity reflects suppressed recombination and more efficient charge separation^48^. Figure 6 presents the PL spectra of Sr@MnO (SM) and La co-doped SrMnO₃ heterostructure nanocomposites (LSM1–LSM4). The SM sample exhibits emission peaks in the 470–550 nm range, attributed to the recombination of photo excited charge carriers under visible light irradiation.

La incorporation significantly quenches PL intensity, especially for LSM3, indicating suppressed recombination and longer charge carrier lifetimes. This behaviour can be attributed to the modification of the electronic structure by La³⁺ ions, which introduce defect states or act as charge trapping centers that facilitate charge separation and suppress recombination processes. Consequently, the availability of photogenerated electrons and holes for surface redox reactions is enhanced, leading to improved photocatalytic activity.

Among all samples, LSM3 exhibits the lowest PL intensity, confirming the most effective suppression of electron–hole recombination. These results are consistent with the photocatalytic degradation performance, where LSM3 shows the highest activity, demonstrating that optimal La doping significantly enhances charge separation and overall photocatalytic efficiency^25,49,50^.

**Fig. 5:**
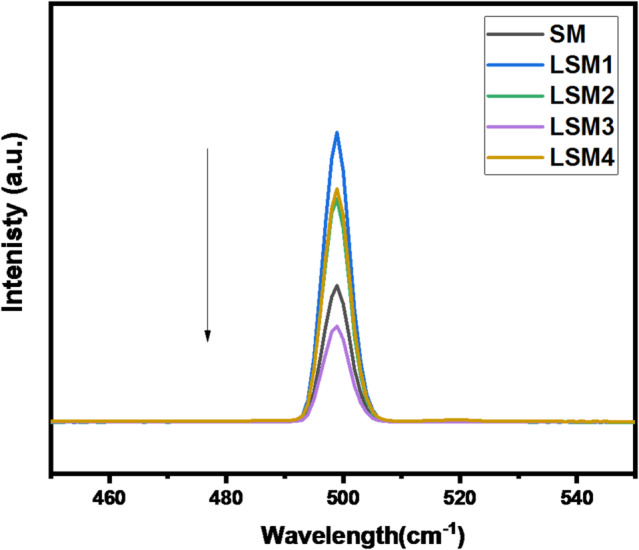
Photoluminescence spectra of SM and LSM nanocomopsites.

#### Photocatalytic activity of La/ Sr@MnO

**Fig. 6:**
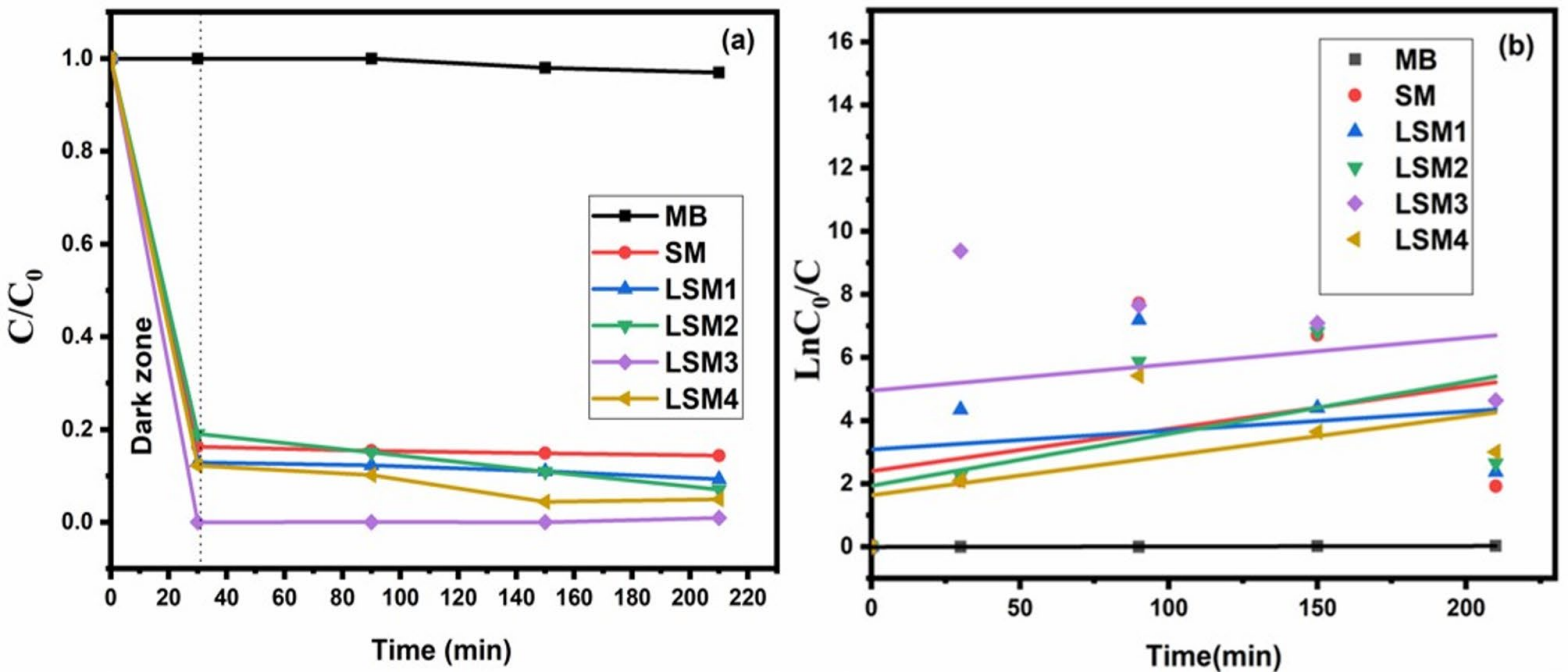
(**a**) The photocatalytic degradation curve and (**b**) Kinetic of MB degradation by SM and LSM nanocomopsites, (catalyst dosage: 1.5 g/L, MB concentration: 20 ppm, natural pH).

The photocatalytic performance of Sr@MnO and La co-doped SrMnO₃ heterostructure nanocomposites with different weight ratios was evaluated for the degradation of methylene blue (MB) dye under visible-light irradiation, as presented in Fig. 6. The results clearly indicate that La incorporation into Sr@MnO significantly enhances the photocatalytic efficiency compared to pristine Sr@MnO. Figure 6(a) illustrates the degradation profile of MB as a function of irradiation time (0, 30, 90, 150, and 210 min). Prior to visible-light exposure, the photocatalyst–MB suspensions were maintained in the dark for 30 min to establish adsorption–desorption equilibrium. The degradation efficiency of MB was calculated using the following equation:

2 $${\text{D }}\% {\text{ }} = {\text{ }}\left( {{\mathrm{C}}_{0}-{\mathrm{C}}/{\mathrm{C}}_{0} } \right)*{\mathrm{1}}00$$

Where C_0_ is the initial MB concentration and C is the remaining concentration at a given irradiation time.

The photocatalytic degradation of methylene blue (MB) under visible-light irradiation shows a significant enhancement upon La incorporation into Sr@MnO heterostructure nanocomposites (LSM). As shown in Fig. 6(a), the pristine Sr@MnO catalyst exhibited a moderate degradation efficiency of 85.6% after 210 min, whereas La doping markedly improved the photocatalytic performance, with LSM3 achieving the highest degradation efficiency of ~ 99.0%. This enhancement can be attributed to dopant-induced self-heterojunctions. In this model, the local variation of La concentration within the SrMnO_3_ crystal creates a built-in electric field. This internal field acts as an ‘electronic interface,’ spatially separating photogenerated electrons and holes within the single-phase lattice. To further understand the reaction mechanism, the degradation kinetics were analyzed using the Langmuir–Hinshelwood (L–H) pseudo-first-order kinetic model, expressed as Eq. (3):

3 $${\mathrm{ln}}\left( {{\mathrm{C}}_{0} /{\mathrm{C}}} \right){\text{ }} = {\text{ k}}_{{\mathrm{a}}} \cdot {\mathrm{t}}$$

Where k_a_ is the apparent rate constant (min^− 1^), C_0_ is the initial concentration (mg L^− 1^), and C is the concentration at irradiation time t. The kinetic data were fitted using a pseudo-first-order model (ln(C₀/C) = k_t_), and the corresponding linear plots are shown in Fig. 6(b). The calculated rate constants (k) and coefficients of determination (R²) are summarized in Table 3, confirming good agreement with the proposed kinetic model. The obtained rate constants were 1.545 × 10⁻⁴, 0.00604, 0.00834, 0.01249, 0.01648, and 0.01342 min⁻¹ for MB photolysis, SM, LSM1, LSM2, LSM3, and LSM4, respectively.

**Fig. 7:**
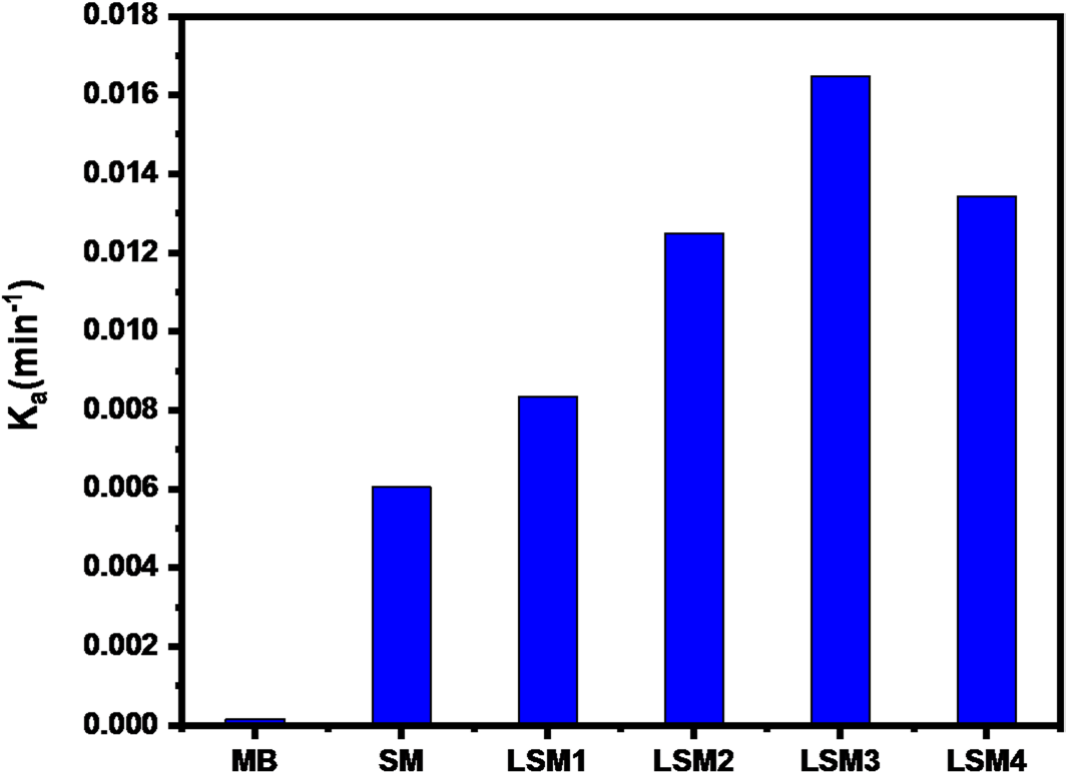
The rate constant of MB, SM, and LSM nanocomopsites (catalyst dosage: 1.5 g/L, MB concentration: 20 ppm, natural pH).

The kinetic analysis, based on the Langmuir–Hinshelwood pseudo-first-order model, further supports these results. The apparent rate constants (kₐ) follow the order: LSM3 > LSM4 > LSM2 > LSM1 > SM > MB, with LSM3 exhibiting the highest value of 0.01648 min⁻¹, as shown in Fig. 7. This enhancement indicates that an optimal La doping level significantly improves photocatalytic activity by promoting charge carrier separation and suppressing electron–hole recombination. In contrast, the slight decrease observed for LSM4 suggests that excessive doping may introduce recombination centers or structural defects, thereby hindering interfacial charge transfer and reducing photocatalytic efficiency.

The superior performance ofLSM3 can be rationalized by considering both structural and electronic modifications introduced by La doping. First, La³⁺ ions may partially substitute Sr²⁺ in the Sr@MnO lattice, introducing oxygen vacancies that enhance the adsorption and activation of oxygen molecules, thereby generating more reactive oxygen species (ROS). Second, La doping can modulate the band structure by narrowing the bandgap and shifting the absorption edge into the visible region, thus enabling efficient utilization of solar energy. Finally, the synergistic effect of Sr, Mn, and La provides abundant active sites for dye adsorption and degradation^24,51,52^. Overall, the results demonstrate that controlled La doping is an effective strategy to enhance the photocatalytic performance of Sr@MnO heterostructure nanocomposites under visible light. The outstanding degradation efficiency and kinetic performance of LSM3 suggest its potential applicability for the treatment of organic dye pollutants in wastewater.

**Table 3:** Kinetic parameters for MB degradation.

Sample	k (min⁻¹)	*R*²
SM	0.00604	0.985
LSM1	0.00834	0.989
LSM2	0.01249	0.992
LSM3	0.01648	0.995
LSM4	0.01342	0.991

#### Band edge position and photocatalytic mechanism validation

To accurately elucidate the photocatalytic behavior of the La co-doped SrMnO₃ system, both the electronic band structure and defect chemistry must be considered. XRD results confirm the formation of a single-phase perovskite structure, excluding the presence of a conventional multi-phase heterojunction. Therefore, the enhanced photocatalytic activity is attributed to defect-engineered electronic modulation and internal charge separation, rather than classical interfacial heterojunction mechanisms.

The band edge positions of SM and LSM samples were estimated using the electronegativity method, and the results are summarized in Table 2. The valence band (E_VB_) potentials are located between + 2.32 and + 2.39 V vs. NHE, while the conduction band (E_CB_) potentials range from + 0.01 to + 0.08 V vs. NHE. The highly positive VB potentials indicate strong oxidative ability, enabling the direct oxidation of surface hydroxyl groups or adsorbed water molecules to generate hydroxyl radicals (•OH), which serve as primary oxidizing species in the degradation process.

In contrast, the CB potentials are more positive than the standard O₂/•O₂⁻ redox potential (− 0.33 V vs. NHE), indicating that direct reduction of molecular oxygen to superoxide radicals is thermodynamically unfavorable. This excludes a conventional band-to-band reduction pathway and necessitates an alternative mechanism for reactive oxygen species (ROS) generation.

Under visible-light irradiation, the photocatalyst absorbs photons and generates electron–hole pairs (e⁻/h⁺). The photogenerated holes in the VB readily oxidize surface hydroxyl groups to produce •OH radicals. Meanwhile, the photogenerated electrons are not directly capable of reducing O₂ due to the insufficient CB potential. Instead, La³⁺ incorporation induces oxygen vacancies and localized defect states within the band gap, which act as shallow electron traps. These defect states capture photogenerated electrons, suppress recombination, and facilitate their gradual transfer to adsorbed oxygen molecules via a defect-mediated multi-step reduction pathway, leading to the formation of superoxide radicals (•O₂⁻).

This mechanism is strongly supported by experimental observations. The significant quenching of photoluminescence (PL) intensity, particularly for the LSM3 sample, confirms efficient suppression of electron–hole recombination and prolonged charge carrier lifetimes. Furthermore, radical scavenger experiments demonstrate that the addition of benzoquinone (BQ) and isopropanol (IPA) markedly inhibits photocatalytic activity, confirming that •O₂⁻ and •OH are the dominant reactive species, while photogenerated holes also contribute to oxidation.

The superior photocatalytic performance of LSM3 can be attributed to the synergistic effects of optimal La incorporation, which:

- Introduces an appropriate concentration of oxygen vacancies and defect states,
- Enhances charge separation and suppresses recombination (as evidenced by PL),
- Reduces crystallite size (~ 9 nm), increasing surface-active sites and shortening charge diffusion pathways,
- And creates dopant-induced internal electric fields, promoting spatial separation of electrons and holes within the single-phase lattice (self-heterojunction-like behavior).

The slight variation in band edge positions with increasing La content further supports that the enhancement originates from electronic structure tuning and defect engineering, rather than the formation of a distinct heterojunction interface.

Finally, the generated reactive species (•OH, •O₂⁻, and h⁺) act synergistically to oxidize methylene blue into mineralization products such as CO₂ and H₂O.

**Fig. 8:**
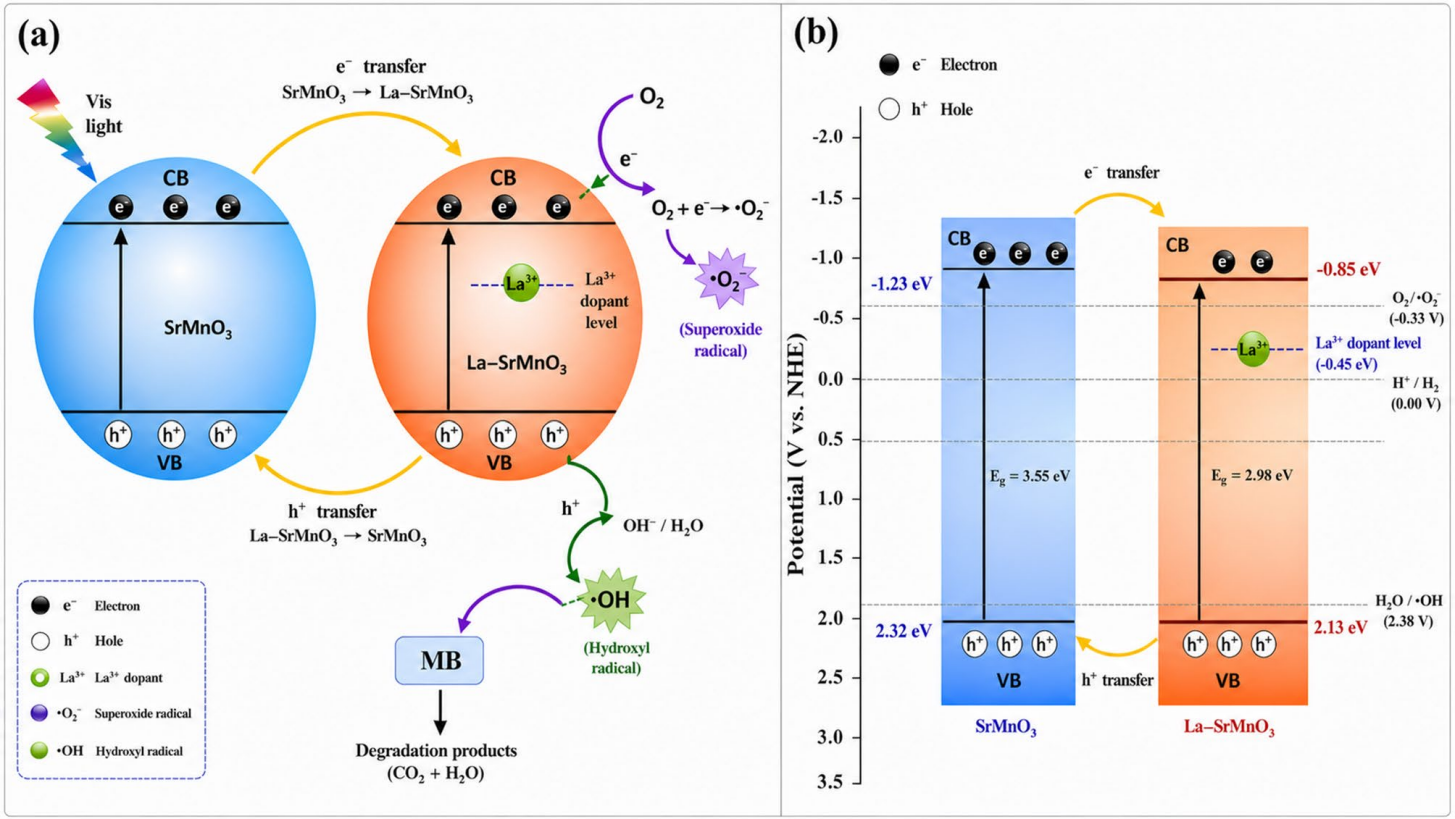
: Schematic photocatalytic mechanism (**a**) and energy band diagram (**b**) of La co-doped SrMnO₃ heterostructure under visible light.

To further elucidate the role of La doping in enhancing photocatalytic efficiency, a schematic energy band diagram illustrating the charge transfer pathways is proposed (Fig. 8 Under visible-light irradiation, the Sr@MnO (SM) and La co-doped SrMnO₃ (LSM) nanocomposites are photoexcited, generating electron–hole pairs according to:

4 $${\mathrm{LSM}} + {{h\nu}} \to ~e_{{CB}}^{ - } + h_{{VB~}}^{ + }$$

Based on the calculated band edge potentials, the valence band (VB) positions (+ 2.32 to + 2.39 V vs. NHE) are sufficiently positive to oxidize surface hydroxyl groups or water molecules, leading to the formation of highly reactive hydroxyl radicals:

5 $$h^{ + } + OH^{ - } ~ \to \cdot {\mathrm{OH}}$$

These •OH radicals act as strong oxidizing agents responsible for the degradation of organic pollutants. In contrast, the conduction band (CB) potentials (+ 0.01 to + 0.08 V vs. NHE) are more positive than the standard reduction potential of O₂/•O₂⁻ (− 0.33 V vs. NHE), indicating that the direct reduction of molecular oxygen by CB electrons is thermodynamically unfavorable. Therefore, the generation of superoxide radicals (•O₂⁻) proceeds via an indirect pathway.

Specifically, La incorporation induces oxygen vacancies and defect states within the band structure, which serve as electron trapping centers:

6 $$e_{{CB}}^{ - } \to e_{{trap}}^{ - } {\text{}}$$

These trapped electrons facilitate stepwise electron transfer to adsorbed oxygen molecules, enabling the formation of superoxide radicals through a defect-mediated process:

7 $$e_trap^{-}+O_2\rightarrow{\cdot}O₂⁻$$

The synergistic effect of defect states and improved charge separation suppresses electron–hole recombination and enhances the availability of reactive species.Finally, the generated reactive oxygen species (•OH and •O₂⁻), together with photogenerated holes, collaboratively oxidize and decompose methylene blue (MB) into harmless mineralization products such as CO₂, H₂O, and inorganic ions:

8 $${\text{MB }} + {\text{ h}}^{ + } \left( {{\mathrm{surface}}} \right)\to {\text{Degradation products}}$$

9 $${\text{MB }} + \cdot {\mathrm{OH}} \to {\text{Degradation products}}$$

10 $${\mathrm{MB}} + \cdot {\mathrm{O}}{} \to {\text{Degradation products}}~$$

The incorporation of La³⁺ ions introduces localized energy levels within the band gap that act as shallow electron traps, thereby facilitating charge separation and suppressing rapid electron–hole recombination, as evidenced by the quenched photoluminescence (PL) intensity observed for the LSM3 sample. The trapped electrons subsequently migrate to the catalyst surface to generate O₂•⁻ radicals, while the holes remain available for •OH formation. Consequently, the enhanced photocatalytic performance is primarily governed by efficient charge separation and reduced recombination, which increase the availability of reactive oxygen species responsible for MB degradation.

Together, these ROS, along with direct oxidation by the holes (h⁺), drive efficient MB degradation.The enhanced photocatalytic activity of La co-doped SrMnO₃ heterostructure arises from the synergistic effects of La incorporation and the Sr@MnO heterostructure, which improve visible-light absorption and promote charge separation. The significantly lower PL intensity of LSM3 indicates suppressed recombination and prolonged carrier lifetimes, enabling more effective redox reactions. While UV–Vis diffuse reflectance spectroscopy (DRS) shows only slight band-gap narrowing, the primary contributors to enhanced photocatalysis are defect-mediated charge transfer and efficient charge separation. Additionally, La doping may create oxygen vacancies that enhance oxygen adsorption and facilitate ROS formation, further accelerating MB degradation. These observations align with kinetic results, where LSM3 exhibits the highest degradation rate constant and photocatalytic efficiency among the studied samples. Overall, MB degradation is primarily driven by •OH and O₂•⁻ radicals, with photogenerated holes also contributing to the oxidation process.

The photocatalytic degradation mechanism and identify the dominant reactive species involved in methylene blue removal, radical scavenger experiments were conducted using specific quenching agents. Isopropanol (IPA), benzoquinone (BQ), and ethylenediaminetetraacetic acid (EDTA) were employed as scavengers for hydroxyl radicals (•OH), superoxide radicals (O₂•⁻), and photo generated holes (h⁺), respectively. The photocatalytic degradation performance was evaluated under identical experimental conditions in the presence of these scavengers.

**Fig. 9:**
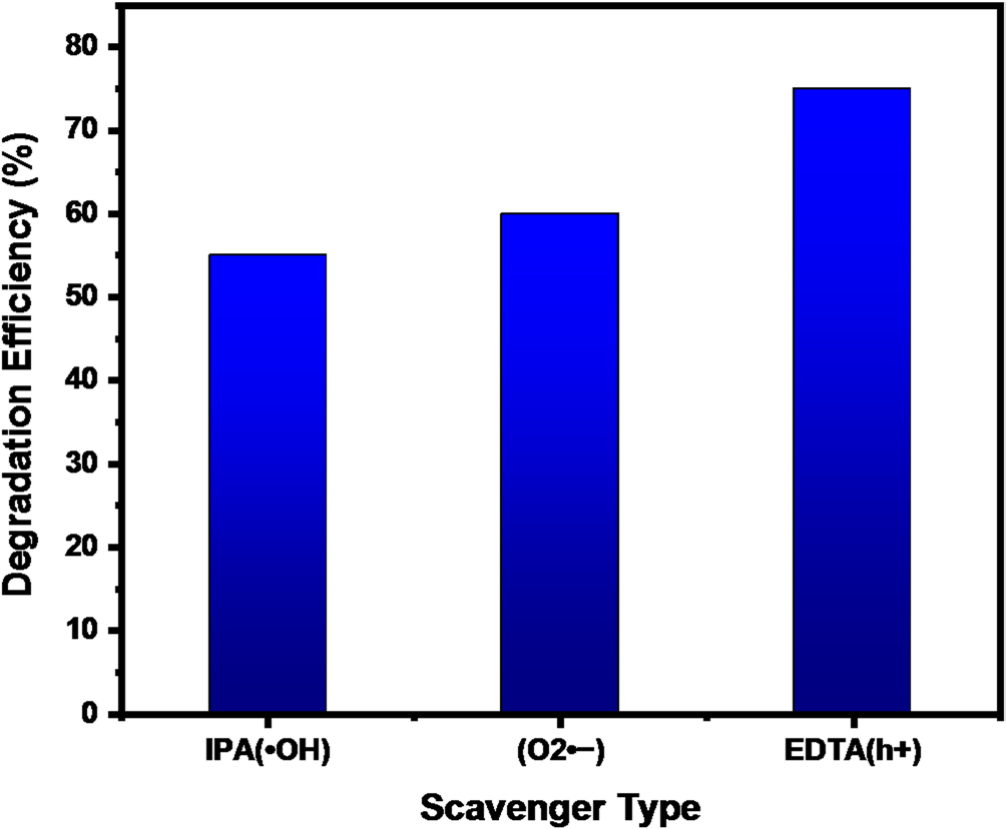
Identification of reactive species during photocatalytic degradation of MB effect of radical scavengers (IPA, BQ, and EDTA) on MB degradation efficiency.

As shown in Fig. 9, the degradation efficiency of methylene blue decreased significantly after the addition of the scavengers compared with the control experiment. In particular, the presence of IPA and BQ resulted in a pronounced suppression of photocatalytic activity, indicating that hydroxyl radicals (•OH) and superoxide radicals (O₂•⁻) are the dominant reactive species responsible for the degradation process. Meanwhile, the addition of EDTA also led to a noticeable reduction in degradation efficiency, suggesting that photogenerated holes (h⁺) contribute to the reaction, although their role is less significant compared with the reactive oxygen species.

To assess the stability and reusability of the prepared photocatalyst, regeneration experiments were conducted using the optimal LSM3 sample over five successive photocatalytic cycles under identical conditions. After each cycle, the catalyst was recovered by centrifugation, thoroughly washed with distilled water and ethanol to remove any residual dye molecules and intermediates, and then dried at 80 °C prior to reuse.

**Fig. 10:**
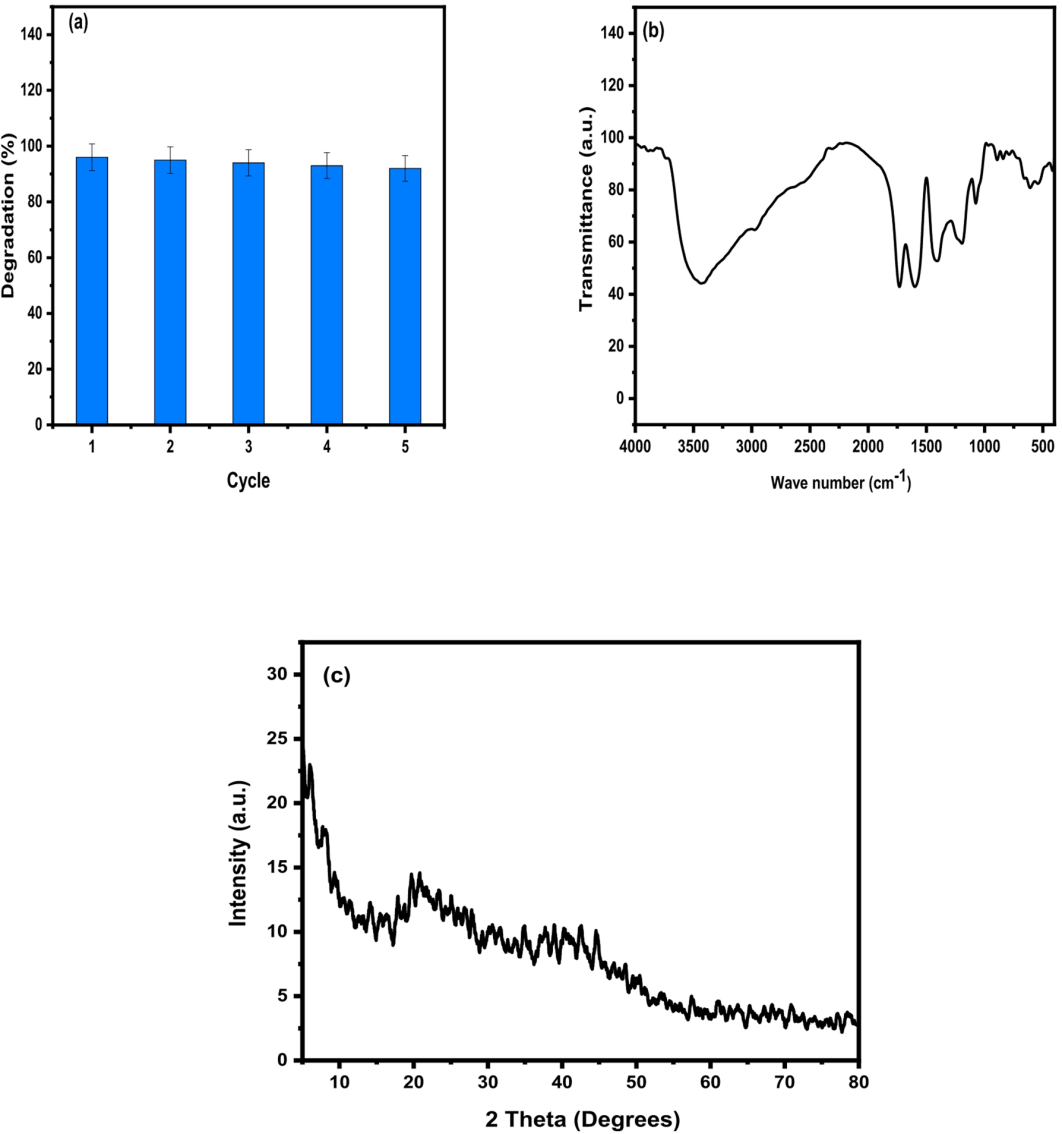
(**a**) Reusability, (**b**) FT-IR and ; (**c**) XRD spectra of LSM3 photocatalyst after photocatalytic degradation for MB.

As illustrated in Fig. 10a, the degradation efficiency showed only a slight decline from 96% in the first cycle to 92% after the fifth cycle, indicating minimal loss of activity. This minor reduction can be attributed to slight catalyst loss during the recovery process or partial surface fouling by intermediate species. Moreover, the FTIR (Fig. 10b) and XRD (Fig. 10c) analyses of the recovered catalyst after the fifth cycle exhibited no noticeable changes in the characteristic peaks, confirming the preservation of its structural integrity.These results demonstrate that the LSM3 photocatalyst possesses excellent stability and reusability, underscoring its potential as an efficient and cost-effective material for practical wastewater treatment under visible light irradiation.

In comparison to the findings of earlier research, Table 4, LSM3 exhibited commendable photocatalytic activity in visible light, as seen in Table 2. La co-doped SrMnO₃ heterostructure nanocomposites in this study outperform many reported systems, with LSM3 achieving ~ 99% degradation and the highest rate constant (0.01648 min⁻¹) under visible light. This enhanced activity arises from La-induced band-gap tuning, improved charge separation, and oxygen vacancy formation, which collectively promote reactive oxygen species generation. These results highlight the dual modification approach with facile strategy that resulte in La co-doped SrMnO₃ as a promising, solar-driven photocatalyst for wastewater treatment.

**Table 4:** **Comparison of photocatalytic efficiency of relevant catalysis with respect to our synthesized** La co-doped SrMnO₃ **NCs.**

Photocatalyst	Light Source	pollutant	Degradation Efficiency (%)	Time (min)	Modification Strategy	Key Feature	Ref.
LaMnO_3_-Ca	Vis-light	MB	68.5	100	A-site doping	Ca substitution at La sites	^53^
LaMnO_3_-Ag	Vis-light	Rose Bengal	92.0	50	Surface modification	Ag nanoparticles on LaMnO₃	^54^
LaMnO_3_ –graphene-Ag	Vis-light	Direct Green BE	97.8	120	Ternary composite	LaMnO₃ + graphene + Ag	^55^
LaMnO_3_	Vis-light	MO	94.7	90	Morphological tuning	Nanostructured LaMnO₃	^56^
LaMnO_3_	Vis-light	MO	90	60	Sol-gel synthesis	Perovskite phase control	^57^
Sr-MnO NPs	UV	acid black1(AB 1) dye in	70	11 h	Sr doping	Sr into MnO nanoparticles	^10^
La_0.7_Sr_1.3_CoO_4_	UV	MO	94	75	A-site co-doping	La + Sr in Ruddlesden-Popper phase	^58^
La_0.7_Sr_0.3_CoO_3_	UVC	MB	75	30	A-site co-doping	La + Sr in perovskite	^59^
Sr-doped ZnO	Vis-light	MB	50	45	Single doping	Sr into ZnO lattice	^60^
X-doped ZnO (X = Sr)	UV	Reactive Red 43	93.43	120	Single doping	Sr into ZnO	^61^
LaMnO_3_	Vis-light	Methylene violet	95	315	Morphological tuning	Shape-controlled LaMnO₃	^62^
Sr@MnO	Vis-light	MB	85.6	210	**Heterostructure formation**	**Sr integrated with MnO**	**This work**
La co-doped SrMnO₃ (LSM3)	Vis-light	MB	99.0	210	**Heterostructure + Doping**	**Sr@MnO + optimal La (1.0%)**	**This work**

## Conclusion

La co-doped SrMnO₃ perovskite nanomaterials were successfully synthesized via a solid-state method and demonstrated enhanced visible-light photocatalytic performance for methylene blue degradation. Structural analysis confirmed the formation of a single-phase perovskite system, while optical studies revealed modest band gap narrowing alongside significantly improved charge separation efficiency, as evidenced by photoluminescence quenching. Among the investigated samples, LSM3 (1.0 wt% La) exhibited the highest photocatalytic activity, achieving ~ 99.03% degradation efficiency with a rate constant of 0.01648 min⁻¹. Band edge analysis indicated that the valence band provides strong oxidative capability for hydroxyl radical generation, whereas the conduction band is insufficient for direct oxygen reduction. Consequently, the photocatalytic process proceeds via a defect-mediated mechanism, where La-induced oxygen vacancies and localized electronic states facilitate electron trapping and enable indirect formation of superoxide radicals.The superior performance of LSM3 is attributed to the combined effects of optimized defect concentration, reduced crystallite size (~ 9.01 nm), enhanced charge separation, and internal electric field–assisted carrier migration within the single-phase lattice. Scavenger experiments confirmed that •OH and •O₂⁻ radicals are the dominant reactive species, with photogenerated holes contributing to the degradation process. Overall, this work demonstrates that defect engineering through controlled rare-earth doping is a highly effective strategy for improving photocatalytic efficiency without relying on conventional heterojunction architectures. The excellent activity and stability of the optimized photocatalyst highlight its potential for practical wastewater treatment applications.

## Data Availability

All data generated or analysed during this study are included in this published article.
